# *Vibrio cholerae* O47 associated with a cholera-like diarrheal outbreak concurrent with seasonal cholera in Bangladesh

**DOI:** 10.1128/msphere.00831-24

**Published:** 2025-04-02

**Authors:** Mohammad Tarequl Islam, Jarin Tasnim, Rabeya Basri, Mohammad Nazmus Sakib, Wali Ullah, Kazi Sumaita Nahar, Abdus Sadique, Marzia Sultana, Eiji Arakawa, Masatomo Morita, Haruo Watanabe, Yann F. Boucher, Anwar Huq, Rita R. Colwell, Munirul Alam

**Affiliations:** 1 International Centre for Diarrhoeal Disease Research, Bangladesh (iccdr,b), Dhaka, Bangladesh; 2 NSU Genome Research Institute, North South University 54495, Dhaka, Bangladesh; 3 Department of Bacteriology, National Institute of Infectious Diseases, Tokyo, Japan; 4 Saw Swee Hock School of Public Health, National University of Singapore 37580, , Singapore; 5 Maryland Pathogen Research Institute, University of Maryland, College Park, Maryland, USA; 6 Institute for Advanced Computer Studies and Department of Cell Biology and Molecular Biology, University of Maryland, College Park, Maryland, USA; University of Wisconsin-Madison, Madison, Wisconsin, USA

**Keywords:** *Vibrio cholerae*, cholera, NOVC, outbreak, genome analysis, serogroup, antimicrobial resistance, genomic island

## Abstract

The Ganges delta of the Bay of Bengal is a recognized hotspot for the emergence and spread of novel variants of *Vibrio cholerae*. Despite being a diverse species, very little information is available concerning environmental and human-associated aspects of *V. cholerae* serogroups, other than the two major epidemic-related serogroups O1 and O139. This represents a crucial gap in understanding the spectrum of diversity, ecology, and epidemiology of the species influencing the dynamics of global cholera. In this study, we describe an emerging variant of *V. cholerae* displaying the antigenic property of serogroup O47, associated with a cholera-like outbreak in coastal Bangladesh where cholera has been endemic for centuries. This outbreak coincides with a rise in cases of cholera caused by *V. cholerae* O1, as well as frequency of isolation of serogroups O47 and O1 from the environment. The *V. cholerae* O47 isolates proved clonal in nature, and their genome biology revealed distinct features, with respect to multidrug resistance (MDR), serogroup-specific genes, genomic island combinations, and overall phylogenetic properties. Genome comparison confirmed the absence of canonical virulence factors of *V. cholerae* O1 and O139, namely, cholera toxin (CTX) and toxin-co-regulated pili (TCP), and the presence of putative virulence factors including type 3 secretion system (T3SS) and an MDR pseudo-compound transposon, carrying genes for macrolide resistance and extended spectrum beta-lactamase. Results of the study suggest that *V. cholerae* O47 could represent an emerging *Vibrio* pathogen with the potential to spread virulence and antimicrobial resistance traits impacting the management of cholera-like diseases.

**IMPORTANCE:**

Despite the global insurgence of human diseases caused by Vibrios in recent years, most research focuses only on the O1 serogroup of *V. cholerae*, leaving a significant gap concerning the environmental and human-associated aspects of other serogroups found in nature. Although other serogroups are often found associated with sporadic diarrhea cases, in 1992–1993, a massive cholera-like diarrhea epidemic was initiated by a “non-O1” serogroup, namely, O139 that temporally displaced O1 from endemic cholera in the Bay of Bengal villages of Bangladesh and India, highlighting the potential threat they might pose. This study describes yet another emerging variant of *V. cholerae*, displaying the antigenic property of serogroup O47, associated with a cholera-like outbreak in a coastal locality in Bangladesh. Findings of the study offer critical insights into the genome biology of *V. cholerae* O47 and its potential implications for understanding their ecology and epidemiology of cholera-like diseases.

## INTRODUCTION

*Vibrio cholerae*, causative agent of the diarrheal disease cholera, is the most famous member of the diverse family *Vibrionaceae*. Even though cholera is a global problem, the Ganges delta of the Bay of Bengal (GDBB) remains a hotspot for the emergence of new pathogenic variants of *V. cholerae* with the potential to impact the epidemiology of cholera at pandemic scales (1, 2). Of the seven recognized cholera pandemics, the currently ongoing seventh pandemic is reported to have originated and spread in distinct waves from the GDBB, propelling global cholera dynamics (3). In the environment, *V. cholerae* exists as a diverse species, with more than 200 serogroups displaying remarkable diversity in its O-antigen (4, 5). Sporadic cases and minor outbreaks of gastrointestinal disease have been traced to various *V. cholerae* genotypes, but cholera pandemics historically are linked with strains of a single phylogenetic lineage, most of which display the O1 antigen on their cell surface.

In the 1990s, a heretofore unknown “non-O1 serogroup of *V. cholerae*, designated O139 Bengal, emerged in the GDBB as an epidemic agent of cholera-like disease, affecting multiple countries of the region (6). The extent of the spread of cholera due to the new serogroup was such that at one point, serogroup O139 was thought to have been causing the eighth pandemic, epidemiologically displacing O1 El Tor (7). *V. cholerae* possessing serogroups other than O1 and O139, collectively referred to as non-O1/O139 *V. cholerae* (NOVC), are known to cause diarrheal cases of sporadic but clustered patterns in different parts of the world (8–11). Alarmingly, a large number of NOVC isolates from different serogroups like O37, O75, and O141 isolated from human infections encode major virulence factors (cholera toxin [CTX] and toxin co-regulated pili [TCP]) and, hence, can cause cholera (12). Moreover, it has been hypothesized that NOVCs can act as reservoirs for some important genetic elements, such as antimicrobial resistance (AMR) genes, and spread them to pandemic generating *V. cholerae* O1 in shared environment (13, 14). AMR traits, as in many other pathogens, have become increasingly relevant to clinical management of the disease. These traits can complicate the treatment and potentially provide a fitness advantage to circulating *V. cholerae* isolates, impacting the ecology and evolution of this pathogen (15).

Despite decades of research, it is still unclear how species and subspecies-level interactions affect emergence, evolution, and spread of novel variants of the cholera bacterium across geographic boundaries. Understanding the spectrum of intraspecies diversity circulating in the GDBB region is crucial to monitor the emergence of novel pathogens and effects of climate, environmental, and population changes. Prevalence, phylogeny, and ecology of NOVCs are factors poorly understood due to several reasons, one of which is the lack of information and genome representation in public databases. Even though NOVC associated with human infections is being reported increasing from different parts of the world in recent years (13, 16, 17), NOVC as a disease-causing agent often goes undetected or underreported in cholera-endemic zones like Bangladesh, where *V. cholerae* O1 causes severe outbreaks (18–20). In this study, we investigated an outbreak of cholera-like disease in rural coastal Mathbaria, Bangladesh, caused by strains of an emerging *V. cholerae* O47 serogroup. Strikingly, the rise in clinical O47 cases was co-incidental with both a rise in O1 cases and in its presence in the environment. The genomic, phylogenetic, and resistome properties of the *V. cholerae* serogroup O47 provided insight as to how its emergence was related to the epidemiology and evolution of cholera-like disease in this region.

## RESULTS AND DISCUSSION

### Co-occurrence of an outbreak of O1 and a group of non-conventional *V. cholerae* in a choleraendemic region

**Fig 1:**
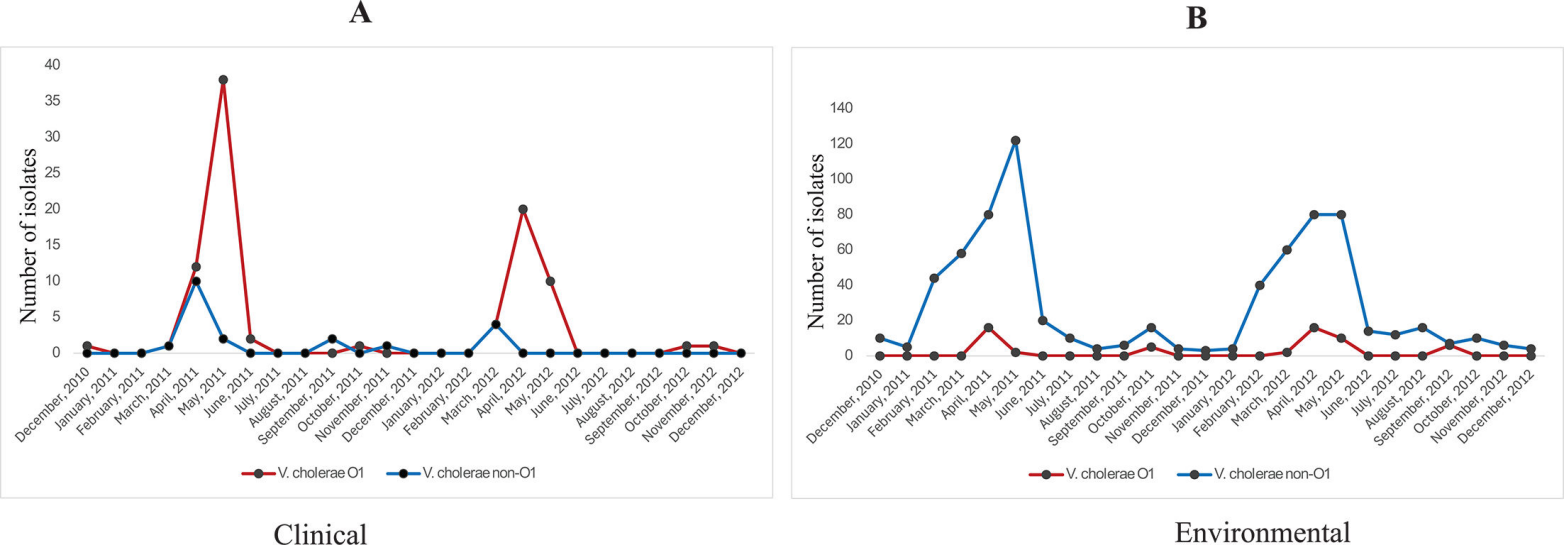
Isolation history of *V. cholerae* O1 and NOVC by month from clinical (A) and environmental (B) samples collected in Mathbaria from December 2010 to December 2012. *X*-axis denotes. *X*-axis denotes the month, and *Y*-axis denotes the cumulative number of isolates from culture-based techniques in bi-weekly sampling scheme

Mathbaria is located in the coastal belt of the Bay of Bengal where tidal water inundates drinking water sources, hence impacting the prevalence of cholera in the area (21). Patterns of seasonal outbreaks of cholera in this region show that the disease usually peaks in the months of April and May (21, 22). Bay of Bengal villages in Bangladesh and India are historical hotspots for cholera, where recurrent seasonal disease often turns into epidemics. In 1992, *V. cholerae* serogroup O139 synonym Bengal initiated a massive cholera-like outbreak by temporarily overtaking the O1 serogroup as the principal cause of endemic cholera in Bangladesh. A later study of *V. cholerae* in Mathbaria, between 2003 and 2007, showed that seasonal cholera in 2005 was initiated by O139 Bengal, forming a small outbreak peak followed by the O1 seasonal outbreak peak (22). Between 2010 and 2017, as part of routine surveillance to study the ecology and epidemiology of *V. cholerae* associated with human disease in coastal Bangladesh, presumptive *V. cholerae* isolates from both clinical and environmental samples were analyzed, using a comprehensive sampling scheme (https://www.gtfcc.org/research/epidemiology-and-ecology-of-v-cholerae-in-bangladesh/). To determine whether serogroups other than O1 also produced outbreaks, retrospective analysis of the samples was undertaken. Inspection of reported clinical cases revealed the start of the O1 seasonal outbreak of cholera in 2011 and 2012 coincided with a peak from a smaller cholera-like outbreak caused by *V. cholerae* isolates that did not agglutinate with O1 or O139 antisera (Fig. 1A). Indeed, in the spring of 2011, a small outbreak of cholera-like diarrhea occurred in Mathbaria just before the usual cholera peak, where the associated bacterium was *V. cholerae*, but phenotypic (Table S1) and molecular assays confirmed it to be NOVC.

PCR screening targeting potential virulence genes revealed that the NOVC isolates (*n* = 12) were negative for canonical *V. cholerae* O1 virulence factors, namely, CTX and toxin-co-regulated pili (*tcp*) (Table 1), which are also negative for zonula occludens toxin (zot) and accessory cholera enterotoxin (ace) found associated with *ctx* (23, 24). Nevertheless, they possessed putative virulence factors presumably contributing to clinical manifestation of diarrhea. All isolates carried the putative virulence-associated genes *toxR*, *hlyA*, *ompU*, and HA/P. Two isolates possessed genes encoding type 3 secretion system (T3SS), and three isolates possessed the gene coding for cholix toxin. These genetic factors have been reported for NOVC isolates associated with cholera-like disease in previous studies (25–28).

**TABLE 1:** Virulence and drug resistance-related traits of NOVC isolates associated with cholera-like diarrhea in Mathbaria, 2011–2012^*a*^

No. of isolates	Yr of isolation	Serogroup	Virulence and resistance traits																
			*ompW*	*toxR*	*rfbO1-ctx-tcpA*	*ompU*	*rtxA*	*rtxC*	*hlyA*	NAG-ST	HA/P	Cholix	T3SS	Pulsotype	Resistance pattern	MIC μg/mL (AZM)	MIC μg/mL (CIP)	*mphA*	*qnrVC*
7	2011	*V. cholerae* O47	+	+	−	+	+	+	+	−	+	−	−	I	AZM-CIP-E-TE-AMP-SXT	2	1.5	+	+
2	2011	*V. cholerae* O9	+	+	−	+	+	+	+	−	+	+	+	II	E-AMP	0.38	0.002	−	−
1	2012	*V. cholerae* O128	+	+	−	+	+	+	+	−	+	+	−	Untypeable	AMP	0.5	0.003	−	−
2	2012	*V. cholerae* O184	+	+	−	+	+	+	+	−	+	−	−	III	E-AMP	0.5	0.38	−	−

^*a*^ +, presence of the gene; −, absence of the gene.

Although NOVC strains can cause sporadic diarrhea, they have rarely been isolated from clinical cases in cholera-endemic villages such as Mathbaria (22, 29), where the major etiological agent of cholera outbreaks is toxigenic *V. cholerae* O1 (Fig. 1A). However, NOVC are frequently isolated from environmental samples, while *V. cholerae* O1 is only rarely found in such samples using culture-based methods (22) (Fig. 1B). Cholera seasonality in coastal Bangladesh has been elucidated in a few studies, indicating a general bimodal pattern of cholera (1, 2). Data on isolation from clinical and environmental samples in December 2010 to December 2012 showed that *V. cholerae* O1 and NOVC, indeed, demonstrated a bimodal pattern in Mathbaria (Fig. 1A and B). Considering the importance of the event of NOVC being associated with clinical cholera and coinciding with seasonal cholera, the serogroup of the *V. cholerae* isolates (*n* = 11) associated with the outbreak was determined at the reference laboratory of the National Institute of Infectious Diseases (NIID), Japan, using available antisera against all known serogroups of *V. cholerae*. Results, shown in Table 1, revealed that seven of the isolates belonged to serogroup O47, two to serogroup O9, and one each to serogroups O84 and O128, respectively. This is the first report of these serogroups being associated with an outbreak of diarrheal disease.

### Molecular typing reveals clonal nature and MDR traits of the outbreak isolates

For initial molecular subtyping of the isolates, pulsed-field gel electrophoresis (PFGE), which was considered the gold-standard molecular typing tool for *V. cholerae* until recently (30, 31), was done. PFGE analysis revealed a serogroup-specific clonal pattern for the O9 (*n* = 2) and O47 isolates (*n* = 7), indicating they were potential outbreak agents (Fig. S1). Remarkable features of the O9 serogroup were presence of T3SS system genes, a potent virulence factor reported to be associated with disease comparable to typical cholera (25). However, for O47 isolates, the outstanding genetic property was not T3SS but its MDR traits (resistant to more than two classes of antibiotics). Susceptibility to common antimicrobials of all NOVC isolates showed that the *V. cholerae* O47 were MDR, with resistance to azithromycin (AZM), ciprofloxacin (CIP), erythromycin (E), tetracycline (TE), ampicillin (AMP), and sulfamethoxazole/trimethoprim (SXT). The other NOVC isolates were sensitive to most of the drugs tested (Table 1). AZM was the drug of choice to treat cholera-like diarrhea at that time in this region (32); hence. resistance to AZM had significance from a clinical perspective. Resistance to AZM had only rarely been reported for *V. cholerae* O1 at that time, and the drug was used extensively to treat cholera or cholera-like diarrhea (32, 33). *V. cholerae* O1 from Mathbaria isolated in the same time period did not show resistance to AZM and CIP (29). MDR *V. cholerae* O47 isolates were screened for known genetic determinants of resistance, and only the O47 isolates were found to contain efflux pump gene *mph*A, which is associated with reduced susceptibility to macrolide antibiotics (E and AZM) (Table 1). Additionally, *qnrVC* gene and mutations in *gyrA* (S83) and *parC* (S85), associated with fluoroquinolone (CIP) resistance in enteric pathogens (34, 35), were detected (Table S3 ).

### Emergence and evolution of MDR *V. cholerae* O47

Considering the importance of the emergence of an MDR *V. cholerae* serogroup causing human disease at the same time that *V. cholerae* O1 was present in the cholera-endemic area, three O47 isolates were selected for whole-genome sequencing (Table S2). The sequenced genomes were compared with those of reference *V. cholerae* O1, O139, and NOVC isolates (Table S4), to identify potential genetic traits related to emergence and evolution as an outbreak agent.

**Fig 2:**
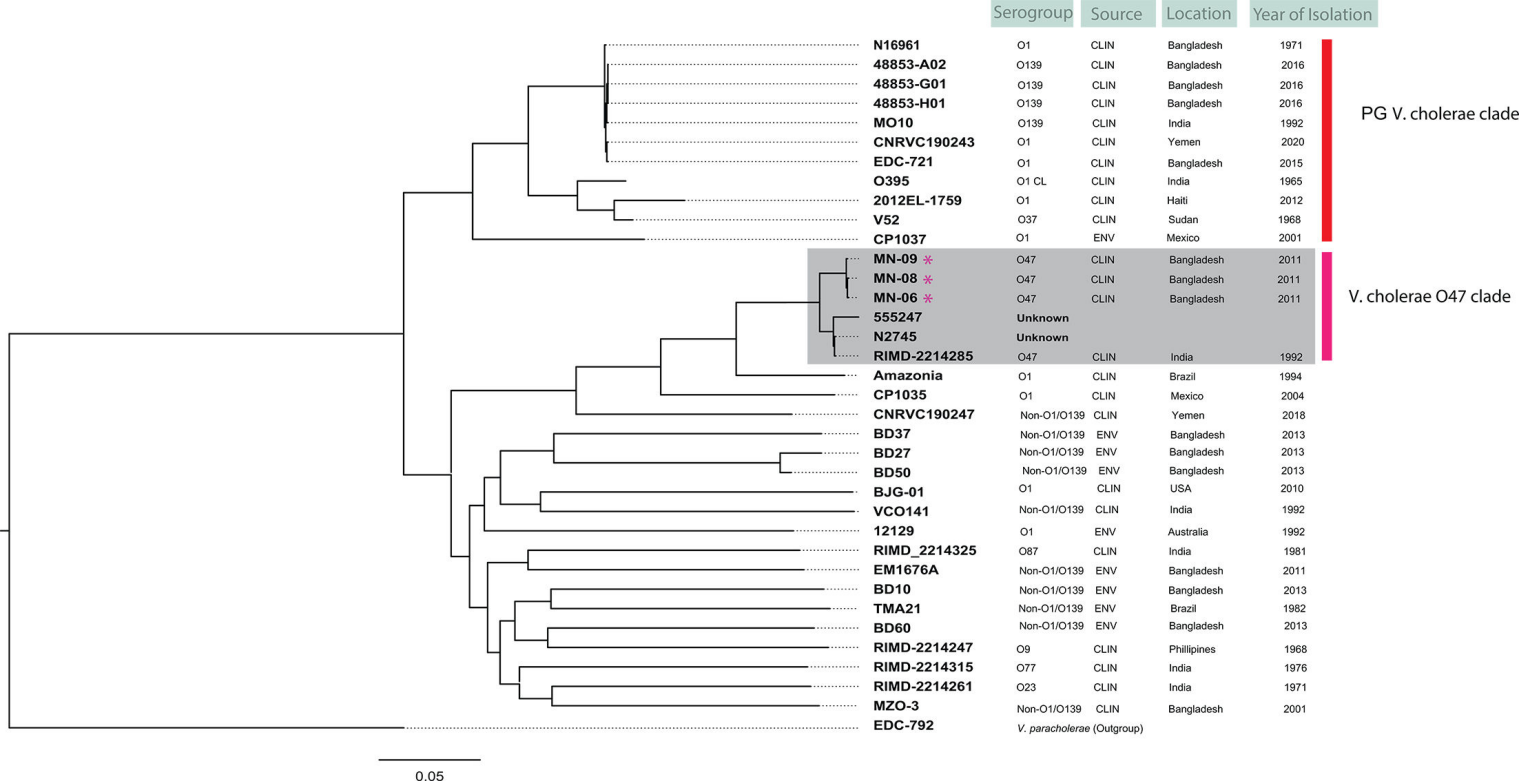
Single nucleotide polymorphism (SNP)-based phylogeny of *V. cholerae* O47. Pandemic-generating (PG) and *V. cholerae* O47 clades are marked by colored bars. The tree was constructed from SNPs called against the *V. cholerae* El Tor reference genome N16961, and a *V*. paracholerae (EDC-792) was used as an outgroup to root the tree. The scale bar represents the number of substitutions per site per genome.

Comparative genomics confirmed the absence of canonical virulence factors of *V. cholerae* O1 and O139, namely, CTX andTCP, in *V. cholerae* O47 strains and the presence of putative virulence factors and several important genomic islands (Table 1). In the core genome phylogeny, NOVC isolates were polyphyletic, and O1/O139 isolates were grouped in a monophyletic cluster. Mathbaria *V. cholerae* O47 strains formed a monophyletic clade with reference *V. cholerae* 047 isolated in India during 1992, which was distinct from other serogroups (Fig. 2). The phylogenetic signal suggested emergence of the O47 serogroup in the clinical setting of Mathbaria in 2011, not a recent seroconversion event, since the genetic backbone of Mathbaria O47 isolates appeared to be similar to the 1992 O47 isolate from India. It is important to point out that recent gain and loss of mobile genetic elements (MGEs) may have played a role in the evolution of the outbreak agent.

Average nucleotide identity of the O47 isolates was >99%, whereas outside the serogroup, the value ranged from 97% to 98% with other *V. cholerae* strains. Digital DNA-DNA hybridization (dDDH) (36), a gold-standard criterion for defining species, showed noticeable distinction of the O47 strains. Within the O47 clade, pairwise comparison of strains yielded dDDH values ranging from 98.7% to 99.9%, whereas it was 81% to 90% for O47 strains with *V. cholerae* isolates outside the clade. The observed coherence between genome data and serogroup-specific properties should be especially useful for detecting human pathogenic *V. cholerae* serogroups such as O47 in the environment. All Mathbaria *V. cholerae* O47 isolates were of the same sequence type (ST558) by traditional multilocus sequence type (MLST) typing (Table S5), while the Indian O47 isolate had near identical sequence type ST623 (one SNP in *pyr*H). cgMLST, which takes all the core genes into consideration, has been shown to have higher resolution in typing *V. cholerae* than the seven gene MLST scheme (37).

### Acquisition of genomic islands and evolution of MDR *V. cholerae* O47

Conservation of certain gene clusters (GCs) suggests phenotypic and perhaps ecological distinctness of the *V. cholerae* O47 isolates, with respect to *V. cholerae* O1, O139, and other NOVC serogroups. Based on comparison of four genomes (from isolates with a confirmed serogroup) of *V. cholerae* O47 with reference genomes, we were able to deduce 11 GCs (GC-1 to GC-11) that could be significant in defining traits and perhaps ecology of the clade.

**Fig 3:**
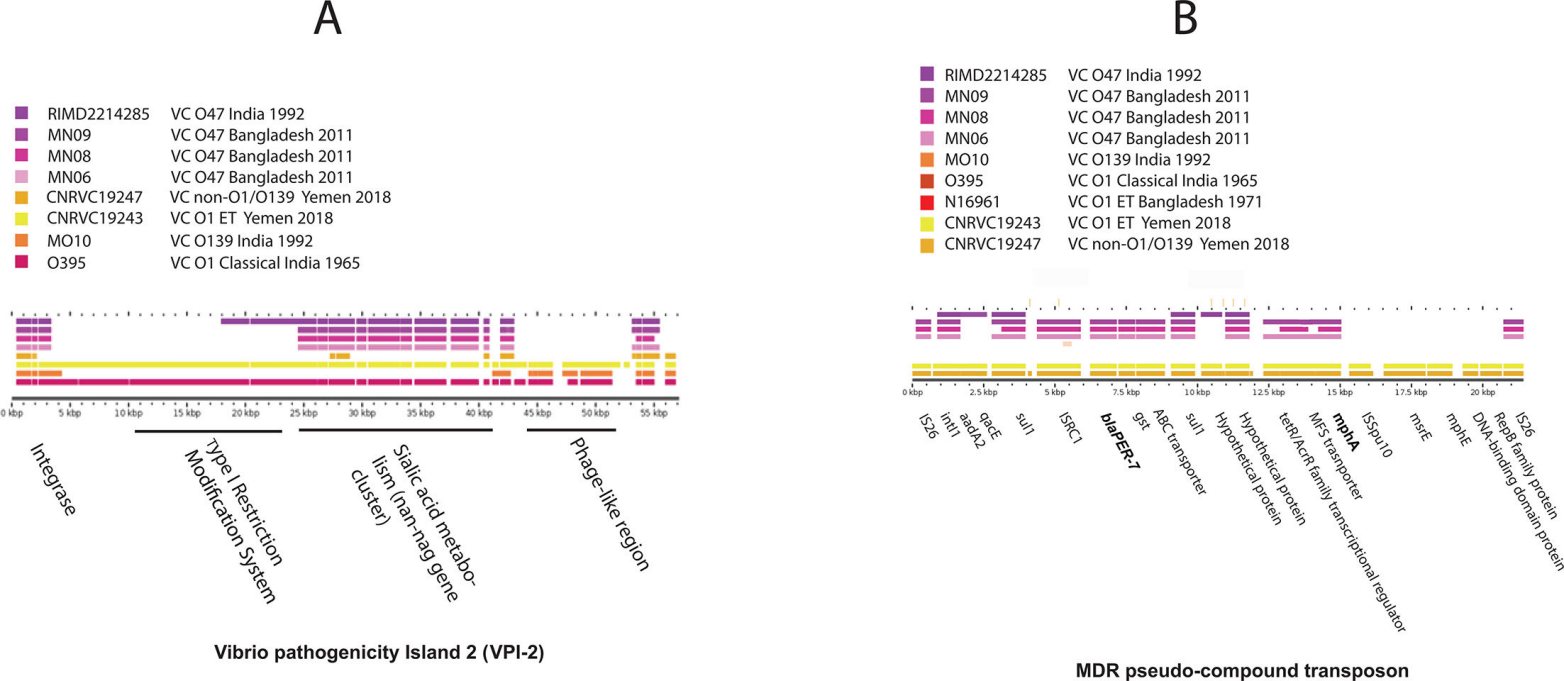
Comparison of gene content in (A) VPI-2 and (B) MDR pseudo-compound transposon found in *V. cholerae* O47 genome in respect to reference genomes. (A) VPI-2 region from reference *V. cholerae* El Tor strain N16961 and for panel B, MDR pseudo-compound transposon found in Yemen *V. cholerae* El Tor strain CNRVC19243 was used as reference.

GC-1 encodes for a ~9-kb putative maltose utilization operon (Fig. 2), which has not been found in *V*. cholerae O1 or O139 but is found sporadically in *V. cholerae* NOVCs and its close sister species *Vibrio paracholerae* and *Vibrio metoecus* (38). GC-2 encodes for a resistance-nodulation-division (RND) family efflux pump with potential utility for AMR, including resistance to macrolides (39). GC-3 encodes a version of *Vibrio* pathogenicity island-2 (VPI-2) found in pandemic *V. cholerae* and thought to be essential for causing human disease (i.e., cholera). Interestingly, all O47 isolates contained the nan-nag GC, related to sialic acid metabolism, found to be part of VPI-2. Sialidase metabolism has been inferred as crucial for adaptation and pathogenesis of Vibrios in the human gut (40). The presence of this GC may be an important contributor to human association and disease progression. VPI-2 has been proposed as a contributing factor for success of pandemic *V. cholerae* (1, 41). This genomic island is consistently present in all biotypes and serogroups associated with cholera worldwide. While all four *V. cholerae* O47 isolates harbored a truncated version of VPI-2 containing the nan-nag region, Mathbaria *V. cholerae* O47 isolates (Fig. 3A) lacked the two gene DNA defense module (VC01770-VC01771) associated with elimination/destabilization of plasmids in seventh pandemic *V. cholerae* (42). GC-4 encodes the O-antigen biosynthetic region present in all *V. cholerae* but displays significant variation in terms of number and type of protein-coding genes. *V. cholerae* O47 isolates have potential serogroup-specific regions found only in this clade and discussed in further detail in a later section. GC-5 designates a putative type 2 secretory pathway, present in all *V. cholerae* O47 isolates but absent in other reported *V. cholerae* isolates from Bangladesh. GC-6 encodes a mannose-mannan utilization GC. This island is rare in *V. cholerae*, and only two other serogroups contain it: O45 and O51, according to the NCBI database. Utilization of mannan has not been reported in *V. cholerae* to date. However, the island is common in the *Vbrio fluvialis* and *Vibrio furnissii* genomes, suggesting horizontal gene transfer in shared ecosystems, perhaps with other niche adaptive traits.

GC-7 encodes a maltose-neopullulanase GC, recently described in sister species *V. cholerae* and *V. paracholerae* (38). GC-8 encodes an outer membrane chitoporin and GC-9 a Tn7 transposon. GC-10 encodes an incC plasmid-like region, reported in *V. cholerae* as well as other enteric bacteria and potentially involved in AMR. Homologs of this region were found in *Salmonella enterica* (Table 2), and deduction of its exact role warrants further investigation. GC-11 encodes GCs including a putative repeat in toxin (RTX) leukotoxin system not found in *V. cholerae* O1 but found sporadically in NOVC (43).

**TABLE 2:** Major genomic islands of *V. Cholerae* O47 in comparison to *V. cholerae* O1, O139, and sister species *V. paracholerae^a^*

GC	Size (bp)	Reference genome accession	Locus in reference genome	Predicted/putative function
Maltose utilization GC (GC-1)	9607	*V. paracholerae* LS997868.1	SAMEA104470976_02944-SAMEA104470976_02949	Maltose utilization
RND efflux pump (GC-2)	4522	*V. paracholerae* LS997868.1	SAMEA104470976_01045-SAMEA104470976_01047	Antimicrobial (macrolide) resistance
Nan-nag GC (GC-3)	12845	*V. cholerae* O1 El Tor CP028827.1	VC01087-VC1769	Sialidase
O-antigen biosynthetic GC (GC-4)	54052	*V. cholerae* O1 El Tor CP028827.1	VC02595-VC02634	O-antigen biosynthesis
Ribose transporter (GC-5)	12154	*V. cholerae* O51 RIMD 2214289 AP023380.1	VCSRO51_0200-VCSRO51_0207	Sugar transport
Mannan utilization island (GC-6)	27958	*V. cholerae* O51 RIMD 2214289 AP023380.1	VCSRO51_2915-VCSRO51_2934	Mannan utilization
Maltose neupullulanase GC (GC-7)	13746	*V. paracholerae* LS997868.1	SAMEA104470976_03366-SAMEA104470976_03377	Maltose utilization
Outer membrane porin (GC-8)	1833	*V. paracholerae* LS997868.1	SAMEA104470976_01796-SAMEA104470976_01797	Chitin utilization
Tn7 transposon (GC-9)	4070	*V. cholerae* O96 RIMD 2214334 AP023385.1	VCSRO96_2236-VCSRO96_2238	AMR
Inc plasmid (GC-10)	24070	*S. enterica* CP129384.1	ETE97_22620-ETE97_22745	AMR
RTX leukotoxin cluster (GC-11)	19181	*V. cholerae* O51 RIMD 2214289 AP023380.1	VCSRO51_1299-VCSRO51_1300	Leukotoxin

^*a*^ Locus positions of predicted islands in reference genomes (determined by BLAST identity N16961 (*V. cholerae* O1), NCTC30 (*V. paracholerae*), RIMD2214289 (*V. cholerae* O51), RIMD 2214334 (*V. cholerae* O96), and *S. enterica* CP129384.1 were used for size calculation and deduction of functions from annotated genes

**Fig 4:**
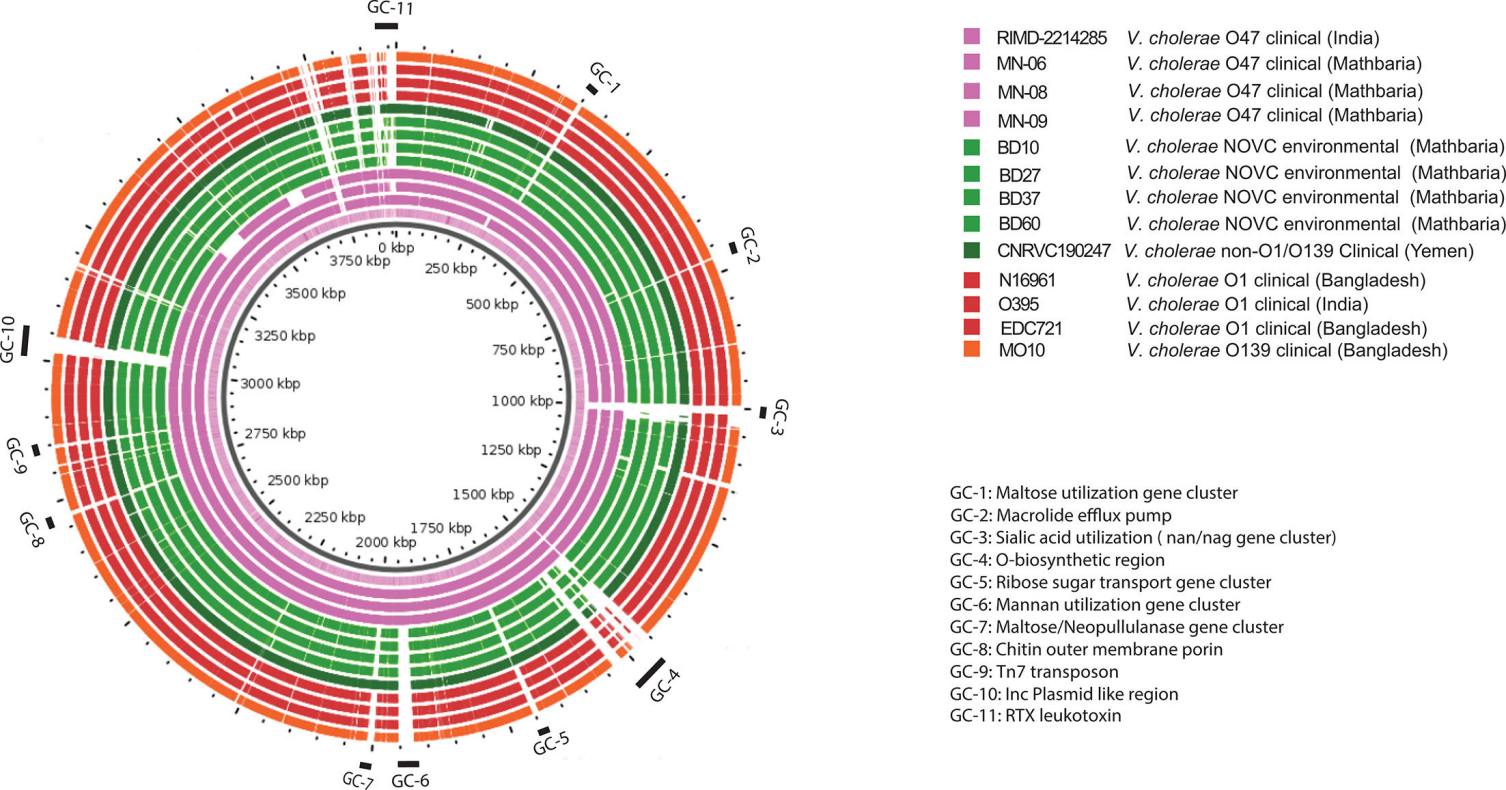
Genome blast atlas comparison of *V. cholerae* O47, *V. cholerae* O1, *V. cholerae* O139, and other NOVC. Each ring in the atlas represents a genome with two chromosomes merged and colored by the chosen color representing a group of organisms. GCs are denoted by black bars, and predicted annotations are mentioned (described in Table 2) in the bottom right panel.

Ecological structuring of *V. cholerae* at the subspecies level has come into light in a recent study suggesting that certain lineages (ecotypes) of *V. cholerae* may have habitat or niche preference distinct from other lineages of the species (44). MGEs can be crucial in enabling a lineage with a property that assists adaptation and niche differentiation and may act as major driving force for the evolution of pathogenic *V. cholerae*, as suggested in recent studies (45–47). The 11 GCs described (GC-1 to GC-11) were conserved in all four *V. cholerae* O47 genomes, perhaps playing an important role in the emergence of the serogroup as a human pathogen. In addition, a remarkable genetic element present in the three Mathbaria O47 isolates sequenced, but not in reference O47 strain RIMD-2214285 from India, is a putative IS transposon-like element showing an MDR phenotype (Fig. 3B). This element has just recently been reported in *V. cholerae* O1 and NOVC and hypothesized to be associated with prolonged cholera outbreaks in Yemen, a country where the seventh cholera pandemic has continued, with a record number of cholera cases (48, 49). A homolog of the element was found in NOVC isolate CNRVC190247 in Yemen during upsurge of the epidemic in that country (49) and denoted an MDR pseudo-compound transposon, carrying genes for macrolide resistance (*mph*A, *mph*E, and *msr*E), extended spectrum beta-lactamase (*bla*PER-7), quaternary ammonium compound (*qac*), sulfonamide resistance (*sul1*), and aminoglycoside resistance (*aadA2*). Mathbaria O47 isolates possess a derivative of the element carrying the entire set of these AMR genes, except *mph*E and *msr*E (Fig. 4). Acquisition of this pseudo-compound transposon likely plays a key role in the MDR phenotype observed in *V. cholerae* O47 isolates associated with diarrhea in Mathbaria.

Emergence of MDR in *V. cholerae* generally is governed by acquisition of MGEs that often carry and spread AMR genes within the population across species and subspecies boundaries. Indeed, emergence of O139 in the 1990s as a pandemic agent coincided with the acquisition of an integrative conjugative element carrying genes coding for resistance to several antibiotics (50). Recent genome-based analysis of worldwide outbreaks and transmission waves shed light on the notion that these MGEs might also have significant adaptive roles, other than drug resistance, hence changing the course of an outbreak (3, 49). In Mathbaria, the AMR pattern of the *V. cholerae* O47 isolates was dissimilar to that of the *V. cholerae* O1 population. Specifically, AZM resistance has not been reported in Bangladesh until 2005 (51), even though AZM has been the drug of choice to treat symptomatic cholera and cholera-like diarrhea (32, 33) over the past decade. Interestingly, AMR surveillance of *V. cholerae* O1 isolates (*n* = 97) from the year 2010–2014 revealed that only one isolate in 2014 isolated showed similar MIC value of 2.0 for AZM, whereas all seven VC O47 isolates from 2011 showed the MIC value for AZM (29). In recent years, reduced susceptibility and resistance toward AZM has been reported commonly in *V. cholerae* O1 (29, 52). This suggests *V. cholerae* O47 as a potential gateway for the transmission of AMR traits to *V. cholerae* O1 in Mathbaria, underscoring the indirect effects that this group might have on the epidemiology of cholera.

### O-antigen biosynthetic region to track an emerging pathogen

**Fig 5:**
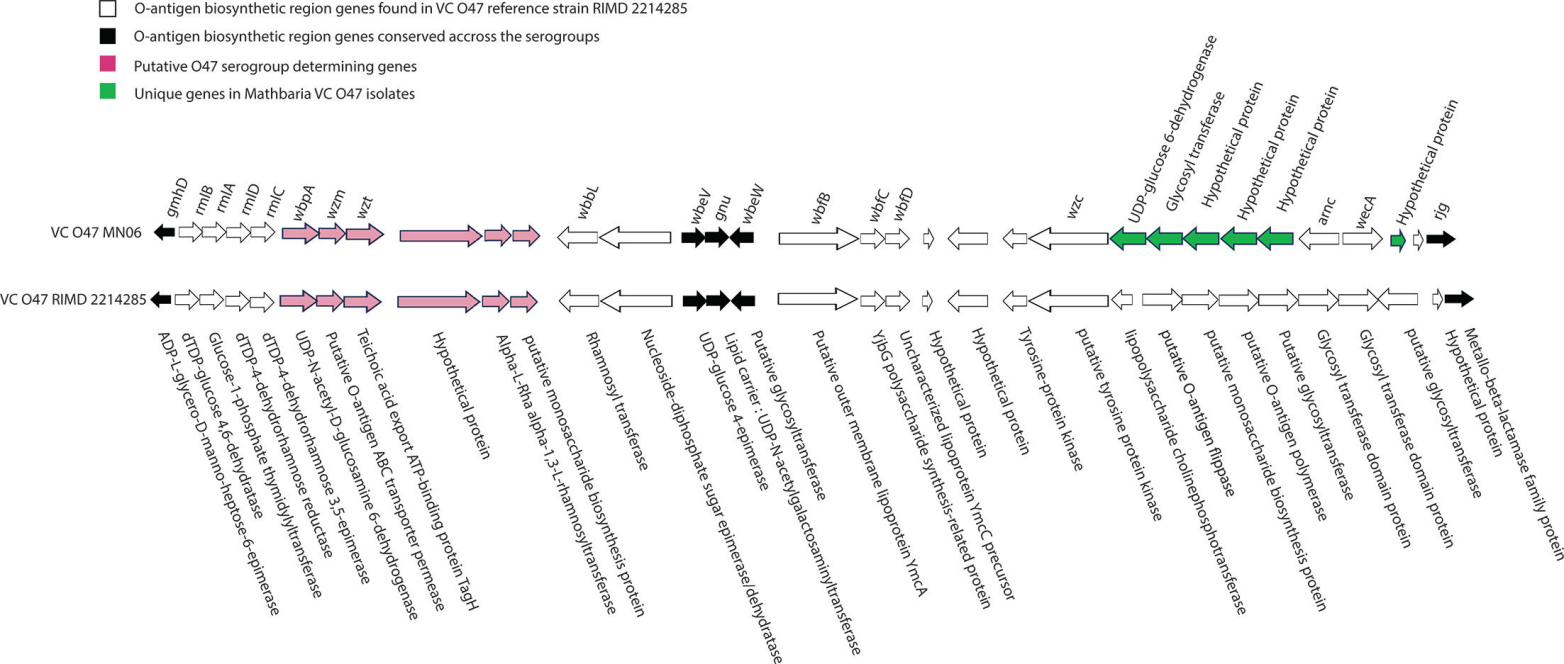
Comparison of the O-antigen-encoding region (wb*) of *V. cholerae* O47 MN06 isolated from Mathbaria outbreak and reference *V. cholerae* O47 RIMD2214285. The genetic orientation organization of the genes is denoted by arrows. Genes conserved across *V. cholerae* serogroups are marked by black arrows, genes that were only found in O47 serogroup are marked by pink arrows, and unique genes only found in Mathbaria outbreak isolates are marked as green arrows.

It has been hypothesized that the genetics of the O-antigen region might have a direct or indirect role in acquisition of the canonical virulence factors (CTX and TCP) and virulence adaptive traits (53). *V. cholerae* O47 isolates have distinct regions in their O-antigen biosynthetic cluster that may define the serogroup-specific structure. The four O47 isolates have six genes not found in *V. cholerae* genomes of serogroups other than O47; hence, this is potentially a serogroup-determining region for *V. cholerae* O47 (Fig. 5). These include UDP-N-acetyl-D-glucosamine 6-dehydrogenase (EC 1.1.1.136) termed *wbp*A; dTDP-4-dehydrorhamnose 3,5-epimerase (EC 5.1.3.13) termed *wzm*; glucose-1-phosphate thymidylyltransferase (EC 2.7.7.24) termed wzt; alpha-L-Rha alpha-1,3-L-rhamnosyltransferase (EC 3.2.1.40), a gene encoding a putative monosaccharide biosynthesis protein; and a hypothetical protein with no functional annotation to date. Interestingly, two isolates that designated NOVC in public databases (N2745 and 555247, Fig. 2) showed 100% homology to the gene set. We hypothesize that these two strains may demonstrate serological characteristics similar to Mathbaria *V. cholerae* O47. Indeed, MLST of those two isolates are identical to *V. cholerae* O47 of this study, and, in genome phylogeny, they belong to the distinct O47 clade. This observation supports the idea that serogroup-specific genes may serve effectively as markers for serogroup identification in ecological studies in the future.

### Conclusion

Emergence of drug-resistant *V. cholerae* O47 as the cause of an outbreak of gastrointestinal disease in cholera-endemic Mathbaria at the start of seasonal cholera caused by *V. cholerae* O1 is an important public health event, with respect to the ecology, evolution, and epidemiology of cholera and cholera-like diseases. The spread of antibiotic determinant genetic elements further highlights the potential role of heretofore unrecognized *V. cholerae* serogroups in cholera scenarios. Distinct genetic properties of *V. cholerae* O47 offer potential markers for tracking this potential pathogen and for establishing a unified tracking strategy of non-conventional *V. cholerae* serogroups as possible agents contributing to larger scale disease. One limitation of the study is the lack of contemporary and contextual data on this emerging pathogen. Development of an effective molecular screening scheme using the data provided in these studies will foster better understanding of such neglected yet important lineages of potentially pathogenic Vibrios.

## MATERIALS AND METHODS

### Bacterial isolation and identification

*V. cholerae* isolates were isolated from stool samples collected from diarrheal patients admitted to the Mathbaria health complex—a rural hospital in Mathbaria (longitude 22.2920° N and latitude 89.9580° E) in Pirojpur, Bangladesh, approximately 400 km southwest of Dhaka—during regular surveillance conducted by icddr,b from December 2010 to December 2012. Isolation and primary identification of *V. cholerae* were performed according to established culture-based and molecular techniques, as described elsewhere (54). Briefly, stool samples were collected in 100-mL stool cups and were transferred to alkaline peptone water broth for enrichment. After 6 h of enrichment, 5–10 µL of enriched broth were streaked onto thiosulfate citrate bile sucrose agar and taurocholate tellurite gelatin agar and then incubated at 37°C for 18–24 h. Presumptive colonies were subcultured on gelatin agar, and *V. cholerae* colonies were tested to determine their serogroups using slide agglutination with polyvalent antiserum, followed by monoclonal and VC serogroup O1- and O139-specific antisera. Template DNA was prepared from the isolated colonies of presumptive *V. cholerae* by boiling method and analyzed by a species-specific PCR for *ompW* gene and a multiplex PCR for detection of *wbe*, *wbf*, and *ctxA* sequences specific for O1 and the O139 serogroups of VC, respectively (22, 54–56). Isolates positive for *ompW* PCR but negative for *wbe* and *wbf* were designated as NOVC.

Serogroup of selected NOVC that showed MDR phenotype or presence of virulence genes was determined in the reference laboratory in Japan using 206 polyclonal O antisera against all known serogroups of *V. cholerae* according to the protocol developed at the NIID (Tokyo, Japan) (57). Data for contemporary *V. cholerae* O1 isolates were gathered from Rashed et al. and Ceccarelli et al. (29, 58) for the comparison purposes.

### Antibiotic susceptibility assay

Antimicrobial susceptibility was determined using standard disc diffusion assay performed on Mueller–Hinton agar plates with 16-h incubation in 35°C temperature according to Bauer et al. and the Clinical and Laboratory Standards Institute guideline (59, 60). NOVC isolates were tested for susceptibility toward AMP (10 µg), SXT (25 µg), mecillinam (25 µg), E (15 µg), AZM (15 µg), TE (30 µg), nalidixic acid (30 µg), CIP (5 µg), imipenem (10 µg), levofloxacin (5 µg), ceftriaxone (30 µg), chloramphenicol (30 µg), cefixime (5 µg), cefepime (30 µg), gentamicin (10 µg), and aztreonam (30 µg), using commercial antibiotic discs (BD BBL SensiDisc, MD, USA). Resistance toward AZM and CIP was subjected for the MIC test using E-test strips (Biomeuriex, Germany), and CLSI breakpoints were used to interpret the results (60).

### Determination of molecular traits

Putative virulence factors were screened using specific primers for *ace*, zot, *tox*R, outer membrane porin (*omp*U), hemagglutinin protease (HA/*P*), hemolysin (*hly*A)*,* RTX (*rtx*A and *rtx*C), T3SS genes (*vcsJ2*, *vspD*, *vcsVUQ2*, *vcsRTCNS2*, *vttRA*, *vttRB*, and *vopF*), and cholix toxin gene (*chx*A) according to conditions described elsewhere (8, 25, 26, 61). PCR for genes related to macrolide resistance were performed according to the protocol described by Sutcliffe et al. (62). Genes related to fluoroquinolone resistance: *gyr*A, *par*C, and *qnr*VC, were detected using a protocol described elsewhere (35), and sequence type was determined using Sanger dideoxy termination nucleotide sequencing. PFGE was performed on selected NOVC isolates following protocol described previously (63).

### Whole-genome sequencing

The genomes of selected *V. cholerae* isolates were subjected for whole-genome sequencing as described previously (38). Genomic DNA was extracted from the cultured isolates using DNeasy Blood and Tissue Kit (Qiagen, Germany) following manufacturer’s instruction. From the extracted genomic DNA, sequencing libraries were prepared using Nextera XT DNA Library Preparation Kit (Illumina, San Diego, CA, USA) according to standard Illumina protocol. Whole-genome sequencing was performed in Illumina MiSeq platform (2 × 250-bp paired-end reads). Quality control of the reads and *de novo* assembly were performed in Geneious Prime 2024.0.4 (64) using default parameters.

### Comparative genomics and phylogenetic analysis

Reference *V. cholerae* genome sequences were extracted from GenBank and used for comparison. A whole-genome SNP analysis was performed based on 2,469,520 shared positions using CSI phylogeny v1.4 and *V. cholerae* N16961 as reference (65). Phylogenetic trees were visualized in FigTree and iTol (66). The genome sequences were annotated in rapid annotation and subsystems technology (2.0) server (67). Genome comparison for BLAST atlas of the genomes and genomic islands was executed in GView server (https://server.gview.ca/). The MLST of the isolates was determined using the seven housekeeping gene scheme for *V. cholerae* listed in pubMLST (37). AMR profile was checked using ResFinder (68).

## Data Availability

Nucleotide sequence data generated in this study are available in the GenBank database under accession numbers SAMN43111401, SAMN43111402, and SAMN43111403 under BioProject number PRJNA1146769.

## SUPPLEMENTAL MATERIAL

Figure S1 msphere.00831-24-s0001.pdf PFGE profile of *V. cholerae* O47 and O9 associated with cholera-like outbreak in Mathabari, Bangladesh.

Table S1 msphere.00831-24-s0002.docx Phenotypic characteristics of the NOVC isolates from Mathbaria clinical samples.

Table S2 msphere.00831-24-s0003.docx Genome accession information for the isolates used in the study.

Table S3 msphere.00831-24-s0004.docx ResFinder predicted phenotype and associated genetic element found in *V. cholerae* O47 isolates.

Table S4 msphere.00831-24-s0005.docx Genome quality information for the three *V. cholerae* O47 isolates sequenced for the study.

Table S5 msphere.00831-24-s0006.docx Seven gene MLST allele types of O47 isolates.

ASM does not own the copyrights to Supplemental Material that may be linked to, or accessed through, an article. The authors have granted ASM a non-exclusive, world-wide license to publish the Supplemental Material files. Please contact the corresponding author directly for reuse.
