# Supplementary material for: Vibrio cholerae O47 associated with a cholera-like diarrheal outbreak concurrent with seasonal cholera in Bangladesh
Source: mSphere. 2025 Apr 2;10(4):e00831-24. doi: 10.1128/msphere.00831-24 (PMC12039230; doi:10.1128/msphere.00831-24)
Supplement: Figure S1 — PFGE profile of V. cholerae O47 and O9 associated with cholera-like outbreak in Mathabari, Bangladesh. [file msphere.00831-24-s0001.pdf]

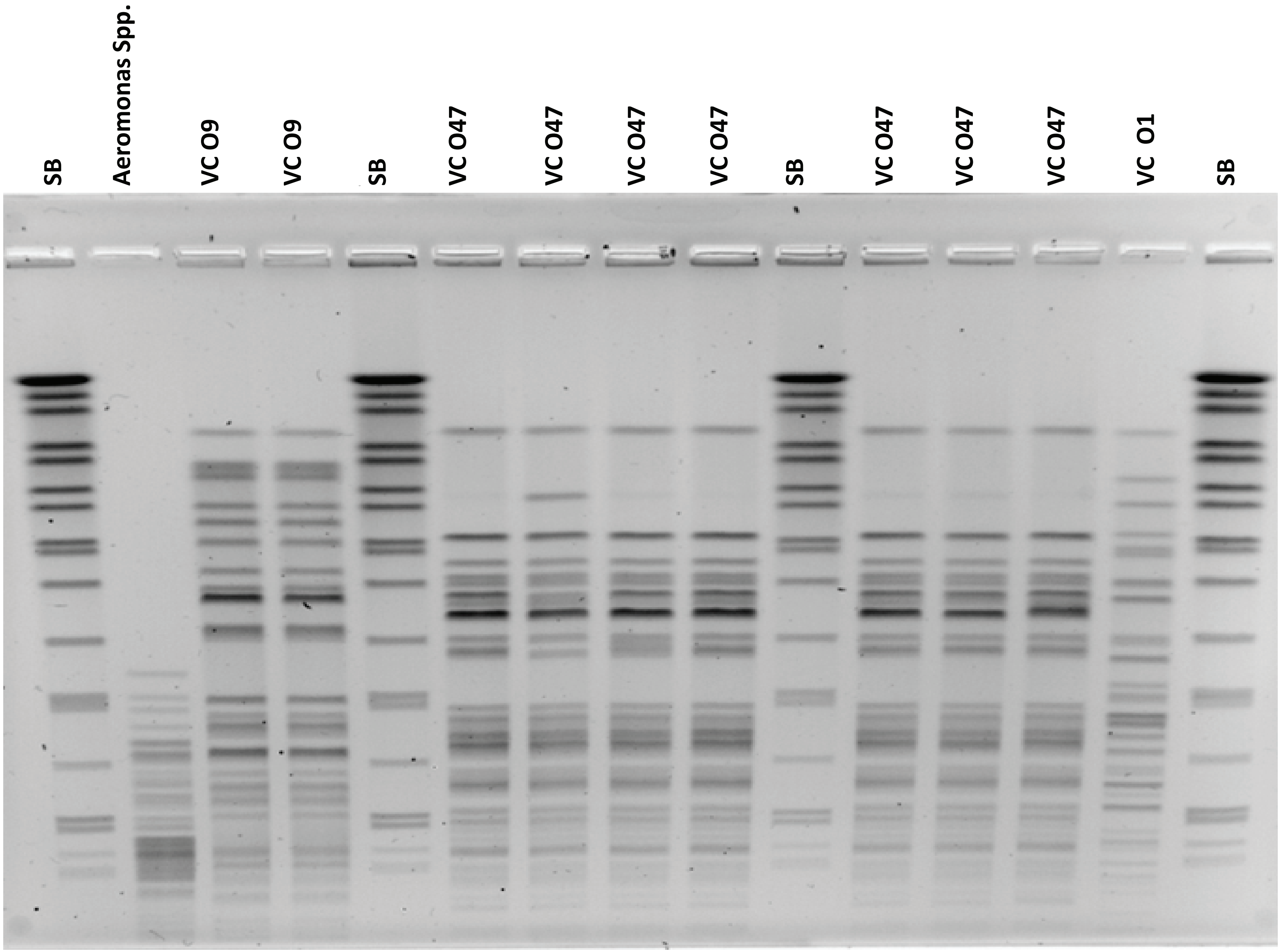

Supplementary figure 1. Pulsed field gel electrophoresis (PFGE) profile of *V. cholerae* O47 and O9 associated with cholera-like outbreak in Mathabari, Bangladesh. For comparison, Aeromonas Spp. isolate in one lane and as size reference *Salmonella braenderup* (SB) was run in quadriplicate lanes.
