# Supplementary material for: Vibrio cholerae O47 associated with a cholera-like diarrheal outbreak concurrent with seasonal cholera in Bangladesh
Source: mSphere. 2025 Apr 2;10(4):e00831-24. doi: 10.1128/msphere.00831-24 (PMC12039230; doi:10.1128/msphere.00831-24)
Supplement: Table S1 — Phenotypic characteristics of the NOVC isolates from Mathbaria clinical samples. [file msphere.00831-24-s0002.docx]

| **Isolate** | **Serogroup** | **Year of Isolation** | **Colony characteristics** | | **Serology (VC polyvalent)** |
| --- | --- | --- | --- | --- | --- |
|  |  |  | **On TCBS Plate** | **On TTGA Plate** |  |
| MN-06 | O47 | 2011 | Yellow | Black centered | - |
| MN-07 | O47 | 2011 | Yellow | Black centered | - |
| MN-08 | O47 | 2011 | Yellow | Black centered | - |
| MN-09 | O47 | 2011 | Yellow | Black centered | - |
| MN-10 | O47 | 2011 | Yellow | Black centered | - |
| MN-11 | O47 | 2012 | Yellow | Black centered | - |
| MN-16 | O9 | 2012 | Yellow | Black centered | - |
| MN-20 | O9 | 2012 | Yellow | Black centered | - |
| MN-22 | O128 | 2012 | Yellow | Black centered | - |
| MN-23 | O184 | 2012 | Yellow | Black centered | - |
| N16961 | O1 | 1971 | Yellow | Black centered | + |

Supplementary table 1. Phenotypic characteristics of the NOVC isolates from Mathbaria clinical samples (2011-2012) in comparison to reference *V. cholerae* O1 N16961
