# Supplementary material for: Vibrio cholerae O47 associated with a cholera-like diarrheal outbreak concurrent with seasonal cholera in Bangladesh
Source: mSphere. 2025 Apr 2;10(4):e00831-24. doi: 10.1128/msphere.00831-24 (PMC12039230; doi:10.1128/msphere.00831-24)
Supplement: Table S2 — Genome accession information for the isolates used in the study. [file msphere.00831-24-s0003.docx]

| **Strain ID** | **Origin** | **Year of Isolation** | **Source** | **Serogroup** | **Accession number** |
| --- | --- | --- | --- | --- | --- |
| N16961 | Bangladesh | 1971 | Clin | *Vibrio cholerae* O1 | [AE003852.1](https://www.ncbi.nlm.nih.gov/nuccore/AE003852.1) |
| 48853_A02 | Bangladesh | 2014 | Clin | *Vibrio cholerae* O139 | LT992492.1, LT992493.1 |
| 48853_G01 | Bangladesh | 2014 | Clin | *Vibrio cholerae* O139 | [LT992486.1, LT992487.1](https://www.ncbi.nlm.nih.gov/nuccore/NZ_LT992487.1/) |
| 48853_H01 | Bangladesh | 2013 | Clin | *Vibrio cholerae* O139 | LT992488.1, LT992489.1 |
| MO10 | India | 1992 | Clin | *Vibrio cholerae* O139 | [AAKF00000000](https://www.ncbi.nlm.nih.gov/nuccore/AAKF00000000) |
| CNRVC-190243 | Yemen | 2018 | Clin | *Vibrio cholerae* O1 | OW443147.1 |
| EDC 721 | Bangladesh | 2015 | Env | *Vibrio cholerae* O1 | [WYCI00000000](https://www.ncbi.nlm.nih.gov/nuccore/WYCI00000000) |
| O395 | India | 1965 | Clin | *Vibrio cholerae* O1 | [CP001235, CP001236](https://www.ncbi.nlm.nih.gov/nuccore/CP001235) |
| 2012EL 1759 | Haiti | 2012 | Env | *Vibrio cholerae* O1 | [JNEW01000000](https://www.ncbi.nlm.nih.gov/nuccore/JNEW01000000) |
| V52 | Sudan | 1968 | Clin | *Vibrio cholerae* O37 | [AAKJ00000000](https://www.ncbi.nlm.nih.gov/nuccore/AAKJ00000000) |
| CP1037 | Mexico | 2003 | Env | *Vibrio cholerae* O1 | ALDB00000000.1 |
| MN09 | Bangladesh | 2011 | Clin | *Vibrio cholerae* O47 | SAMN43111403 |
| MN08 | Bangladesh | 2011 | Clin | *Vibrio cholerae* O47 | SAMN43111402 |
| MN06 | Bangladesh | 2011 | Clin | *Vibrio cholerae* O47 | SAMN43111401 |
| 5552447 | England | 2019 | Clin | *Vibrio cholerae* non-O1/O139 | AAXNXX010000001.1 |
| N2745 | China | Not available | Clin | *Vibrio cholerae* non-O1/O139 | VSGG01000051.1 |
| RIMD 2214285 | India | 1973 | Clin | *Vibrio cholerae* O47 | BOHI01000001.1 |
| Amazonia | Brazil | 1994 | Clin | *Vibrio cholerae* O1 | AFSV01000000 |
| CP1035 | Mexico | 2004 | Clin | *Vibrio cholerae* O1 | [AJRM00000000](https://www.ncbi.nlm.nih.gov/nuccore/AJRM00000000) |
| CNRVC190247 | Yemen | 2018 | Clin | *Vibrio cholerae* non-O1/O139 | OW443150.1, OW443151.1 |
| BD37 | Bangladesh | 2013 | Env | *Vibrio cholerae* non-O1/O139 | QEDP00000000.1 |
| BD27 | Bangladesh | 2013 | Env | *Vibrio cholerae* non-O1/O139 | QEDY00000000.1 |
| BD50 | Bangladesh | 2013 | Env | *Vibrio cholerae* non-O1/O139 | QEDF00000000.1 |
| BJG-01 | USA | 2010 | Clin | Vibrio cholerae O75 | AMWF00000000.1 |
| RIMD 2214379 | India | 1993 | Clin | Vibrio cholerae O141 | BODX00000000.1 |
| 12129-1 | Australia | 1985 | Env | *Vibrio cholerae* O1 | [ACFQ00000000](https://www.ncbi.nlm.nih.gov/nuccore/ACFQ00000000) |
| RIMD 2214325 | India | 1981 | Clin | Vibrio cholerae O87 | BOIU00000000.1 |
| EM1676A | Bangladesh | 2011 | Env | *Vibrio cholerae* O1 | [APFY00000000.1](https://www.ncbi.nlm.nih.gov/nuccore/APFY00000000.1) |
| BD10 | Bangladesh | 2013 | Env | *Vibrio cholerae* non-O1/O139 | QECS00000000.1 |
| TMA21 | Brazil | 1982 | Env | *Vibrio cholerae* non-O1/O139 | ACHY00000000.1 |
| BD60 | Bangladesh | 2013 | Env | *Vibrio cholerae* non-O1/O139 | QECW00000000.1 |
| RIMD 2214247 | Philippines | 1968 | Clin | Vibrio cholerae O9 | BOIX00000000.1 |
| RIMD 2214315 | India | 1976 | Clin | Vibrio cholerae O77 | AP023383.1, AP023384.1 |
| RIMD 2214261 | India | 1971 | Clin | Vibrio cholerae O23 | BOGL00000000.1 |
| MZO-3 | Bangladesh | 2001 | Clin | *Vibrio cholerae* non-O1/O139 | [AAUU00000000](https://www.ncbi.nlm.nih.gov/nuccore/AAUU00000000) |
| EDC 792 | Bangladesh | 2016 | Env | *Vibrio paracholerae* | [WYCC00000000](https://www.ncbi.nlm.nih.gov/nuccore/WYCC00000000) |

**Supplementary Table 2. Genome accession information for the isolates used in the study.**
