# Supplementary material for: Vibrio cholerae O47 associated with a cholera-like diarrheal outbreak concurrent with seasonal cholera in Bangladesh
Source: mSphere. 2025 Apr 2;10(4):e00831-24. doi: 10.1128/msphere.00831-24 (PMC12039230; doi:10.1128/msphere.00831-24)
Supplement: Table S3 — ResFinder predicted phenotype and associated genetic element found in V. cholerae O47 isolates. [file msphere.00831-24-s0004.docx]

| **Antimicrobial** | **Class** | **WGS-predicted phenotype** | **Genetic background** | ***V. cholerae* O47 Isolates** | | | |
| --- | --- | --- | --- | --- | --- | --- | --- |
|  |  |  |  | **MN-06** | **MN-08** | **MN-09** | **RIMD 2214285** |
| Ciprofloxacin | quinolone | Resistant | qnrVC5 | + | + | + | - |
| Amoxicillin | beta-lactam | Resistant | blaPER-7 | + | + | + | - |
| Amoxicillin+clavulanic acid | beta-lactam | Resistant | blaPER-7 | + | + | + | - |
| Ampicillin | beta-lactam | Resistant | blaPER-7 | + | + | + | - |
| Ampicillin+clavulanic acid | beta-lactam | Resistant | blaPER-7 | + | + | + | - |
| Cefepime | beta-lactam | Resistant | blaPER-7 | + | + | + | - |
| Cefotaxime | beta-lactam | Resistant | blaPER-7 | + | + | + | - |
| Cefoxitin | beta-lactam | Resistant | blaPER-7 | + | + | + | - |
| Ceftazidime | beta-lactam | Resistant | blaPER-7 | + | + | + | - |
| Piperacillin | beta-lactam | Resistant | blaPER-7 | + | + | + | - |
| Piperacillin+tazobactam | beta-lactam | Resistant | blaPER-7 | + | + | + | - |
| Aztreonam | beta-lactam | Resistant | blaPER-7 | + | + | + | - |
| Ticarcillin | beta-lactam | Resistant | blaPER-7 | + | + | + | - |
| Ticarcillin+clavulanic acid | beta-lactam | Resistant | blaPER-7 | + | + | + | - |
| Sulfamethoxazole | folate pathway antagonist | Resistant | sul1 | + | + | + | + |
| Sulfamethoxazole | folate pathway antagonist | Resistant | sul2 | + | + | + | - |
| Trimethoprim | folate pathway antagonist | Resistant | dfrA1, dfrA31 | + | + | + | - |
| Erythromycin | macrolide | Resistant | mph(A) | + | + | + | - |
| Azithromycin | macrolide | Resistant | mph(A) | + | + | + | - |
| Spiramycin | macrolide | Resistant | mph(A) | + | + | + | - |
| Telithromycin | macrolide | Resistant | mph(A) | + | + | + | - |
| Tetracycline, doxycycline | tetracycline | Resistant | tet(A) | - | + | + | - |
| streptomycin | aminoglycoside | Resistant | aadA1 | - | - | - | + |
| spectinomycin | aminocyclitol | Resistant | aadA1 | - | - | - | + |

**Supplementary table 3.** Summary of ResFinder predicted phenotype and associated genetic element found in *V. cholerae* O47 isolates.
