# Supplementary material for: Vibrio cholerae O47 associated with a cholera-like diarrheal outbreak concurrent with seasonal cholera in Bangladesh
Source: mSphere. 2025 Apr 2;10(4):e00831-24. doi: 10.1128/msphere.00831-24 (PMC12039230; doi:10.1128/msphere.00831-24)
Supplement: Table S4 — Genome quality information for the three V. cholerae O47 isolates sequenced for the study. [file msphere.00831-24-s0005.docx]

| **Isolate name** | **Genome size (bp)** | **No. of CDS** | **No. of rRNAs** | **No. of tRNAs** | **G+C content (%)** | **No. of contigs** | **N50 (bp)** | **Coverage** | **Genome accession no.** |
| --- | --- | --- | --- | --- | --- | --- | --- | --- | --- |
| MN-06 | 4,201,254 | 3885 | 25 | 95 | 47.2 | 65 | 148554 | 27X | JBGGTT000000000 |
| MN-08 | 4,147,930 | 3,670 | 11 | 78 | 47.2 | 85 | 129828 | 26X | JBGGTS000000000 |
| MN-09 | 3,929,156 | 3491 | 12 | 124 | 47.8 | 124 | 133669 | 15x | JBGGTR000000000 |

**Supplementary table 4. Genome quality information for the three *V. cholerae* O47 isolates sequenced for the study.**
