# Supplementary material for: Vibrio cholerae O47 associated with a cholera-like diarrheal outbreak concurrent with seasonal cholera in Bangladesh
Source: mSphere. 2025 Apr 2;10(4):e00831-24. doi: 10.1128/msphere.00831-24 (PMC12039230; doi:10.1128/msphere.00831-24)
Supplement: Table S5 — Seven gene MLST allele types of O47 isolates. [file msphere.00831-24-s0006.docx]

| **Isolate** | **Serogroup** | **MLST alleles** | | | | | | |
| --- | --- | --- | --- | --- | --- | --- | --- | --- |
|  |  | ***adk*** | ***gyrB*** | ***mdh*** | ***metE*** | ***pntA*** | ***purM*** | ***pyrC*** |
| MN-06 | O47 | 26 | 5 | 14 | 50 | 31 | 14 | 45 |
| MN-08 | O47 | 26 | 5 | 14 | 50 | 31 | 14 | 45 |
| MN-09 | O47 | 26 | 5 | 14 | 50 | 31 | 14 | 45 |
| RIMD 2214285 | O47 | 26 | 5 | 14 | 50 | 31 | 14 | 123 |
| N16961 | O1 | 7 | 11 | 4 | 37 | 12 | 1 | 20 |
| MO10 | O139 | 7 | 11 | 4 | 37 | 12 | 1 | 20 |

**Supplementary table 5. Seven gene MLST allele types of O47 isolates alongside reference O1 and O139 isolates.**
